# Janus-faced taurine: protection or toxicity?

**DOI:** 10.1186/2193-1801-4-S1-P47

**Published:** 2015-06-12

**Authors:** Andrey Taranukhin, Pirjo Saransaari, Simo Oja

**Affiliations:** School of Medicine, University of Tampere, Finland

**Keywords:** taurine, ethanol, hypoglycemia

Taurine is a simple sulfur-containing amino acid ubiquitously distributed in the tissues of most animals. It is involved in a wide range of physiological processes including osmoregulation, lipid metabolism, intracellular calcium regulation, neuronal development, neuromodulation and cell protection. Using in vivo model of acute ethanol intoxication (5 g/kg) in 7-day-old mice we have found that taurine treatment at total dose 2 g/kg has saved about 50 % of dying neurons from ethanol-induced apoptosis in the internal (Taranukhin et al, 2009, 2010) and the external (Taranukhin et al, 2012) granular layers of developing cerebellum. However, any further increase in taurine doses (4-6 g/kg) aiming to protect more neurons against alcohol-induced apoptosis poses to threat to the whole organism and kills 7-day-old mice thus treated. Since the high doses of taurine alone or ethanol alone did not lead to animal death, lethality appears to be due to ethanol and taurine combined toxicity. We reveal the 50 % and 100 % lethal doses of taurine and ethanol combination for developing (7-day-old), adult (5-6-month-old) and old (12-13-month-old) mice and can conclude that the toxicity of ethanol and taurine combination is age-dependent. A dramatic drop in blood glucose levels observed in 25 % of 7-day-old and 40 % of elderly mice treated with taurine and ethanol suggests that one of the reason of animal death may be hypoglycemia (Taranukhin et al., 2013, 2015). Based on the results obtained we conclude that taurine may have beneficial effects in protecting brain cells against the apoptosis induced by alcohol. However, our finding on the toxicity of combined taurine and ethanol prompts serious concern particularly for young people mixing taurine-containing energy drinks with alcohol.

